# Carbohydrate-Binding Modules of Potential Resources: Occurrence in Nature, Function, and Application in Fiber Recognition and Treatment

**DOI:** 10.3390/polym14091806

**Published:** 2022-04-28

**Authors:** Yena Liu, Peipei Wang, Jing Tian, Farzad Seidi, Jiaqi Guo, Wenyuan Zhu, Huining Xiao, Junlong Song

**Affiliations:** 1 International Innovation Center for Forest Chemicals and Materials and Jiangsu Co-Innovation Center for Efficient Processing and Utilization of Forest Resources, Nanjing Forestry University, Nanjing 210037, China; lyn@njfu.edu.cn (Y.L.); wangpeipei@njfu.edu.cn (P.W.); jingtian@njfu.edu.cn (J.T.); f_seidi@njfu.edu.cn (F.S.); jiaqi.guo@njfu.edu.cn (J.G.); ppzhuwy@njust.edu.cn (W.Z.); 2 Department of Chemical Engineering, University of New Brunswick, Fredericton, NB E3B 5A3, Canada; hxiao@unb.ca

**Keywords:** carbohydrate-binding modules (CBM), classification and configuration, CBM-substrate interactions, CBM conjugate additives

## Abstract

Great interests have recently been aroused in the independent associative domain of glycoside hydrolases that utilize insoluble polysaccharides-carbohydrate-binding module (CBM), which responds to binding while the catalytic domain reacts with the substrate. In this mini-review, we first provide a brief introduction on CBM and its subtypes including the classifications, potential sources, structures, and functions. Afterward, the applications of CBMs in substrate recognition based on different types of CBMs have been reviewed. Additionally, the progress of CBMs in paper industry as a new type of environmentally friendly auxiliary agent for fiber treatment is summarized. At last, other applications of CBMs and the future outlook have prospected. Due to the specificity in substrate recognition and diversity in structures, CBM can be a prosperous and promising ‘tool’ for wood and fiber processing in the future.

## 1. Introduction

Carbohydrate-binding modules (CBMs) are a class of multi-module enzyme proteins and their function is to respond to bind to the carbohydrate substrate [1,2,3]. They usually link to the catalytic domain (CD) that responds to react with the polysaccharide [2,4]. Over 300,208 carbohydrate-binding modules are reported in the Carbohydrate Active enZYmes Database [5] by April 2022, which can be divided into 89 CBM families.

Cellulose-binding domains (CBDs) are the earliest-discovered CBMs which were used to be catergozied based on their sequence homology [6]. However, with the in-depth study of carbohydrate hydrolases, more modules in carbohydrate-active enzymes were discovered that could bind, in addition to cellulose, to other types of carbohydrates such as chitin, glucan, xylan, or starch. Hence, the carbohydrate-binding module (CBM) was used as a more inclusive terminology for reclassifying of these polypeptides [1,7].

Cellulose is the most abundant and widely distributed natural organic polymer in nature, mainly found in plant biomass [8,9,10]. Cellulose is the major component of plant fibers. Fibers are found to serve in many practical applications, such as porous materials [11,12] and paper products. Fibrous porous materials in turn can be used for the fabrication of fiber-reinforced composites [13,14]. In addition, fractal model simulation analysis can help characterize the motion characteristics of fluid in fibrous porous media, thereby understanding the physical mechanism of fluid transport in such media [15]. In the paper industry, the fibers are usually subjected to beating [16] or refining [17] to enhance their bonding capacity to meet the requirements of paper strength. Sometimes, chemicals were used as well [18]. Recently, the secondary fibers have been utilized in paper industry due to production costs and environmental concerns. Indeed, simple mechanical or chemical treatment could not afford the satisfactory strength for paper products made of secondary fibers [19,20,21]. Consequently, fiber treatment research has become increasingly important [22]. Among them, biological treatment attracts the researchers’ attention gradually, and enzyme treatment is one of the popular biological treatment methods [23]. The enzyme treatment is mild and environmentally friendly. The enzymes used in the paper industry include xylanase, laccase, cellulase, and so on [24,25,26]. Cellulase treatment can promote fiber swelling, fibrillation, and foliation, therefore reducing the energy consumption of beating [27]. However, one disadvantage of using the whole cellulase is that the hydrolysis activity of enzymes can reduce the fiber strength [28]. In addition, the cost of cellulase is high and the operation of biological treatment is complex.

CBMs are small in size [29], flexible [30], stable [31], strong in identification [32], with strong plasticity [33], and they can be fused with enzymes or organisms to improve their functions [34]. As one part of cellulase, CBMs are used alone or along with other reagents for improving the fiber properties in recent years [35]. Using CBMs for fiber treatment can avoid losing the fiber strength caused by the hydrolysis activity of CDs of the enzymes. The primary function of CBMs is to aid CDs in improving enzymes’ catalytic efficiency. CBMs can not only bind or interact with polysaccharides [36], but also can be obtained and exist in a pure form [37] which are used as ‘probes’ or ‘channels’. Using the above characteristics, the researchers discovered different types of CBMs and used CBMs as ‘tools’ for substrate recognition, binding, and papermaking. Therefore, the wide range of the application of CBMs, the ways that CBMs recognize, bind, or interact with fibers, and how to use CBMs cost-effectively to solve the problem of insufficient secondary fiber strength have gradually become new research directions.

This mini-review provides the types, potential prospecting sources, structures, and functions of CBMs first. Then, the recent progress on CBMs in fiber recognition and fiber treatment, which is of great significance to the applications of CBMs in the papermaking industry and biological field is surveyed. Finally, the future research and development direction of CBMs are provided.

## 2. CBMs: Classification, Sources, Structures, and Functions

CBMs are widely distributed in nature [38] and are present in enzymes secreted by bacteria, fungi, and archaea [39]. Typical fungi sources are *Trichoderma reesei* [40], *Caldanaerobius polysaccharolyticus* [41], *Rhizopus oryzae* [42] and *Polymyxa* [43]. Fungi have developed to produce a set of glycoside hydrolases (GHs) and oxidoreductive enzymes, the synergistic action of which is required for enzymatic degradation of lignocellulose [44]. Bacteria commonly used in research are *Clostridium thermocellum* [45], *maritima* [46], *Rhodothermus marinus* [47], *bacillus halodurans* [48] and *alcaligenes* [49]. There are other microorganisms containing CBMs, such as *actinomycetes* [50]. Various types of CBMs are obtained from different microorganisms. Additionally, through genetic engineering, different expression vectors are constructed to obtain single or multiple CBMs, and used CBMs for substrate recognition and fiber treatment.

**Figure 1:**
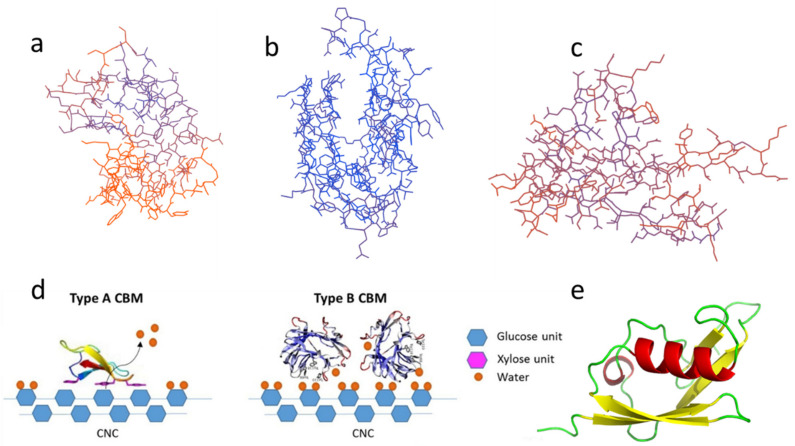
Different types of CBM: (**a**) Type-A, CBM3; (**b**) Type-B, CBM4; (**c**) Type-C, CBM9; (**d**) Schematic of binding of type-A (left) and type-B (right) CBMs on nanocrystalline cellulose (CNC) reprinted from Ref [53] with permission from Elsevier; (**e**) Type-A CBM1 with SUMO solubilizing label. The above structure diagrams are drawn using the base sequences from Table 1 through the Swiss model and Pymol.

There are many ways to classify CBMs. Based on structural, functional similarities and the different ligand binding sites, CBMs can be divided into three types, namely, ‘surface-binding’ CBMs (type-A), ‘glycan-chain-binding’ CBMs (type-B), and ‘small-sugar-binding’ CBMs (type-C) [51]. While according to amino acid sequence similarity and the 3D structure of the adsorption module, CBMs from different sources can be divided into families [52]. Some typical CBMs and their organisms and base sequence are summarized in Table 1 for reference. This table provides a convenient reference for subsequent researchers. Researchers can use this base sequence directly and no longer need to find and identify CBM from the website. For example, desired CBMs can be obtained by genetic engineering (the SUMO nobility tag can be added), as shown in Figure 1e.

### 2.1. Type-A CBMs

Type-A CBMs contain a hydrophobic surface, and the binding of CBMs tends to be distributed in a plane or near a plane, binding to the surface of crystal regions of carbohydrate substrate [54]. A schematic diagram of the binding of type-A CBMs on the fiber substrate is shown in Figure 1d. CBM1 and CBM3 are two typical type-A CBMs. Their 3D configurations are illustrated in Figure 1a [53]. CBM1, the smallest CBM currently found in nature, consists of approximately 36 residues and typically contains two or three disulfide bonds and a plane including three aligned aromatic residues along with several polar residues [55,56,57]. Uppsala University reported the first NMR spectrum of the CBM1 synthesized by solid peptide sequences from the most abundant cellulase in Trichoderma [58].

### 2.2. Type-B CBMs

The crystal structure of type-B CBMs shows that the protein of type-B CBMs often contains grooves or cracks of different depths, which is shown in Figure 1b [59]. They are grooved when the binding sites bind to amorphous cellulose [37] or mannan [60]. The schematic diagram of the binding of general type-B CBMs on the fiber substrate is displayed in Figure 1d [53]. Most type-B CBMs are produced by enzymes secreted by bacteria. The aromatic group only interacts with the free single-chain polysaccharide [45]. The crystal structure of CBM of *cellobiohydrolase A* derived from *Clostridium thermocellum* is the first discovered crystal structure of cellulase CBM4 [61]. And Alahuhta, et al. [62] have solved the X-ray structure of *CelK* CBM4 from *C. thermocellum.*

### 2.3. Type-C CBMs

The typical configurations of type-C CBMs, including CBM9, 14, etc., are illustrated in Figure 1c. Type-C CBMs mainly interact with the end of the polysaccharide chain. Due to steric hindrance, only monosaccharides, disaccharides, trisaccharides, or the terminal sugar group of polysaccharides bind to type-C CBMs [3]. Type-C CBMs was first known from lectins, which are widely found in animals, plants, and microorganisms, and can bind to free sugars in solution. A lectin contains multiple CBMs and can selectively bind to a specific glycosyl [63]. At present, there are few related studies on type-C CBMs.

### 2.4. Other Classification Methods

Other classification methods can be based on the family and folding configuration. In terms of configuration, members of the large majority of CBM families are β-conformations, including β-sandwich, β-Strefoil, Cysteine knot, Unique, OD fold, and Hevein fold [64]. What is interesting is that different types of CBMs can coexist in a single protein, which suggests that current classifications may not cover all functional classifications of CBMs found in nature [65]. And more and more CBMs from different sources are being discovered. The structures, functions, and characteristics of CBMs lay a foundation for CBMs to conjugate or fuse with other polymers and eventually apply in substrate recognition and fiber treatment.

**Table 1:** Summary of the CBMs used for detection and the sequence of different CBMs.

CBM		Organism (Representative Example)	Common Ligands	Sequence	Gene Bank	Ref.
A	1	*Cel6A, Cel7A*	Cellulose, hemicellulose, chitin	Cel6A: ACSSVWGQCGGQNWSGPTCCASGSTCVYSNDYYSQCL Cel7A: TQSHYGQCGGIGYSGPTVCASGTTCQVLNPYYSQCL	AAA34212.1 CAM98445.1	[66]
	3	*A. thermocellum*	Cellulose, chitin	TPTKGATPTNTATPTKSATATPTRPSVPTNTPTNTPANTP VSGNLKVEFYNSNPSDTTNSINPQFKVTNTGSSAIDLSKLTLRYYYTVDGQKDQTFWCDHAAIDLSKLTLRYYYTVDGQKDQTFW QFVEWDQVTAYLNGVLVWGKEHHHHHH	CAP78917.1	[67]
	10	*T. reesei. 7B*	NM	MCNWYGSLTPLCVTTTSGWGYENGKSCV…CNWYGTLYPLCVTTQSGWGWWENSQSCIS	NM	[68]
	20	*β-amylase,* *B. cereus*	Starch, cyclodextrins	TPVMQTIVVKNVPTTIGDTVYITGNRAELGSWDTKQYPIQLYYDSHSNDWRGNVVLPAERNIEFKAFIKSKDGTVKSWQTIQQSWNPVPLKTTSHTSSW	BAA34650.1	[62]
B	4	*Cellulase K Clostridium thermocellum*	Xylan, β-1,3, glucan, β-1,3-1,4-glucan, β-1,6-glucan, amorphous cellulose	NDLLYERTFDEGLCYPWHTCEDSGGKCSFDVVDVPGQPGNKAFAVTVLDKGQNRWSVQMRHRGLTLEQGHTYRVRLKIWADASCKVYIKIGQMGEPYAEYW NNKWSPYTLTAGKVLEIDETFVM	ABN51650.1	[69]
	11	*Endo-β-1,4- glucanase,* *C.fimi;xylanase,* *Rhodothermus Marinus;* *Laminarinase,* *Thermotoga maritima MSB8*	Xylan, β-1,3-glucan, β-1,3-1,4-glucan, β-1,6-glucan and amorphous cellulose	YGEQLIEDFEGAMQWAAYSGVDATASCKISSGKSNNGLEITYAGSSNGYWGVVDNEHRNQDWEKWQ KISFDIKSSNTNEVRLLIAEQSKIEGEDGEHWTYVIKPSTSWTTIEIPFSSFTKRMDYQPPAQDGSETFD LYKVGSLHFMYSNSNSGTLNIDNIKLIGL	ACL75216.1	[47,59]
	17	*Endo-β-1,4-glucanase, C. cellulovorans*	Amorphous cellulose, oligosaccharides	ATPIVQLLRNKGNENLIIVGNPFWSQRPDLAADNPINDSNTMYSVHFYSGTNPISTVDTNRDNAMSNVRYALNHGAAVFATEWGTSLATGTTGPYLAKADAWLDFLNGNNISWCNFSISNKDEKAAALNSLTSLDPGSDKLWADNELTTSGQYVRARIKGAYYATPVDPVTNQPTAPKDFSSGFWDFNDGTTQGFGVNPDSPITAINVENANNALKISNLNSKGSNDLSEGNFWANVRISADIWGQSINIYGDTKLTMDVIAPTPVNVSIAAIPQSSTHGWGNPTRAIRVWTNNFVAQTDGTYKATLTISTNDSPNFNTIATDAADSVVTNMILFVGSNSDNISLDNIKFTK	AAB40891.1	[70]
	44	*Endoglucanase J. Clostridium thermocellum*	Cellulose, xyloglucan, β-glucan, lichenan	SRWKEVKFEKGAPFSLTPDTEDDYVYMDEFVNYLVNKYGNASTPTGIKGYSIDNEPALWSHTHPRIHPDNVTAKELIEKSVALSKAVKKVDPYAEIFGPALYGFAAYETLQSAPDWGTEGEGYRWFIDYYLDKMKKASDEEGKRLLDVLDVHWYPEA	BAA12070.1	[45,71]
	9	*Xylanase A, T.maritima MSB8*	Glucose, cellobiose	56-166: SFEGTTEGVVPFGKDVVLTASQDVAADGEYSLKVENRTSPWDGVEIDLTGKVKSGADYLLSFQVYQSSDAPQLFNVVARTEDEKGERYDVILDKVVVSDHWKEILVPFSPT 205-339: VIYETSFENGVGDWQPRGDVNIEASSEVAHSGKSSLFISNRQKGWQGAQINLKGILKTGKTYAFEAWVYQNSGQDQTIIMTMQRKYSSDASTQYEWIKSATVPSGQWVQLSGTYTIPAGVTVEDLTLYFESQNPT	AAD35155.1	[3]
C	13	*Actinohivin,* *Actinomycete K97;* *Xylanase 10A, S.lividans*	α (1-2)mannobiose/lactose, galactose	ASVTIRNAQTGRLLDSNYNGNVYTLPANGGNYQRWTGPGDGTVRNAQTGRCLDSNYDGAVYTLPCNGGSYQKWLFYSNGYIQNVETGRVLDSNYNGNVYTLPANGGNYQKW	BAA97578.1	[50]
	14	*Chitinase,* *Aedes aegypti, Homo sapiens*	Chitotriose	CTGDGLFPDPDSCKKYYVCSNGHIFEFSCPDGLLFDQQNQICNWPEMVDC	AAZ39947.1	[72]

NM: Not mentioned; Bold indicates the Organisms and Gene Bank corresponding to the sequences in the table.

## 3. Substrate Recognition and Binding by CBMs

The diversity, specificity, and stability of CBMs make them ideal ‘tools’ for studying the structure of cellulose substrate [73]. Therefore, researchers use CBMs as ‘probes’ or ‘channels’ to target, immobilize or interact on substrates. The researchers used modern technology to study the CBMs-substrates interaction. They found that CBMs mainly ‘identify’ substrates through affinity binding between themselves and the substrates.

### 3.1. Substrate Recognition and Binding by CBMs as ‘Probes’

Affinity attachment is of particular interest as it ensures the controlled orientation of the active molecule [74]. Compared with traditional adsorption, affinity attachment has strong adaptability [75], high selectivity [76], and good spatial accessibility [77]. Common biosorptions include bacteria, fungi, etc. CBM is suitable for biosorption because of its enzymatic inactivity, small molecular weight and good stability [78]. As mentioned above, type-A CBMs preferentially bind to crystalline cellulose, type-B CBMs mainly bind to chains, while type-C CBMs mainly bind to smaller oligosaccharides. However, the conditions of binding (pH, activity, temperature, etc.), adsorption capacity, adsorption rate, reusability and economic benefits still need further research. Additionally, the precise mechanism of binding needs to be explored and proved further.

**Figure 2:**
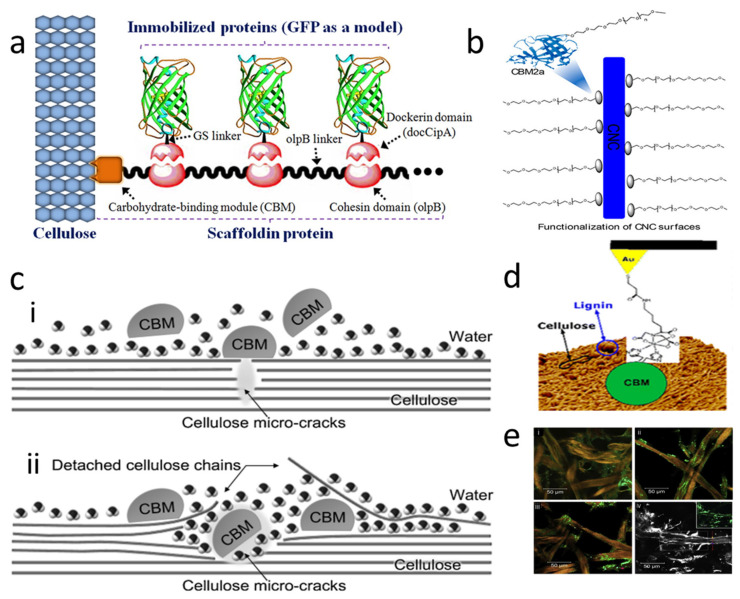
Identification of fibers and substrates by different CBMs. (**a**) Strategies for site-specific and high-loading GFP immobilization on microcrystalline cellulose, reprinted from Ref [81] with permission from American Chemical Society. The GFP−scaffoldin complex could be immobilized on the cellulose surface via CBM−cellulose interaction; (**b**) Schematic representation of a cellulose nanocrystal(CNC) covered with CBM-PEG, reprinted from Ref [87] with permission from American Chemical Society; (**c**) Schematic illustration of morphogenesis of cellulose fibers mediated by CBM1: (**i**) CBM on the surface of cellulose; (**ii**) CBM-promoted amorphization, reprinted from Ref [89] with permission from Elsevier; (**d**) AFM studies different lignin coverage, lignin types of substrates, and the total adhesion of biomass to CBM as a function of surface lignin coverage, reprinted from Ref [95] with permission from American Chemical Society; (**e**) Confocal analysis of interactions between CBMs (labeled with Alexa Fluor 488 C5 Maleimide(Invitrogen)) and filter paper (FP) samples, reprinted from Ref [89] with permission from Elsevier.

Some researchers have taken advantage of the recognition specificity of different types of CBMs to characterize the fiber morphology first [79]. Gao, et al. [80] used CBM17 (type-B) with Mono-Cherry fluorescent protein (CFP-CBM17) and CBM3 (type-A) with Green fluorescent protein fluorescent labels (GFP-CBM3) quantitatively to measure the number of fibers in crystalline and amorphous regions. Guo [70] synthesized fluorescent probes containing GFP-CBM to check the crystallization index of fibers. Li, et al. [81] developed a new immobilization method to simulate the natural cellulosome system. GFP was used as the fixed model specifically bound to the scaffold protein through the cohesion-dockerin interaction, while the scaffold protein was bound to the cellulose through the CBM-cellulose interaction, which is shown in Figure 2a. This mild and simple method could achieve site-specific immobilization, and the maximum load capacity of GFP could reach ~0.508 μmol/g cellulose. Later, Bombeck, et al. [82] discovered a method, named Fluorescently-tagged carbohydrate-binding module (FTCM), which is based on the fluorescent signal from CBMs-based probes designed to recognize specific polymers such as crystalline cellulose, amorphous cellulose, xylan, and mannan. Due to its strong recognition ability and low molecular weight [83], Badruna, et al. [84] used CBMs as probes to explore complex polysaccharide topochemistry in muro and to quantify enzymatic deconstruction. CBM can also be used as a ‘channel’ to connect substrates to some polymers [85,86]. Aissa [87] modified the surface of nanocrystalline cellulose (CNC) through CBM. CBM2a has a strong affinity for crystalline cellulose and is functionalized with acetylene at the terminal amine position, as shown in Figure 2b. And the alkyne group, which was introduced onto the cellulose surface with CBM2a, underwent a Click reaction with polyethylene glycol (PEG) to modify CNC surfaces.

Many researchers are interested in the binding mechanism of CBMs-substrates interactions. Numerous studies have established that three aromatic residues on a CBM surface are needed to bind cellulose crystals and therefore, tryptophans contribute to a higher binding affinity than tyrosines. Orlowski, et al. [88] used multiple, long classical atomic-resolution molecular dynamics (MD) simulations to probe the molecular mechanisms of CBMs and expansin binding to cellulose. Bernardes, et al. [89] found that the addition of CBMs promoted the production of additional reductive ends of cellulase in insoluble substrates. They proved that the binding of CBMs with cellulose was almost heterogeneous and irreversible, as the adsorption of CBMs on the fibers resulted in the amorphization of the fibers, as shown in Figure 2c. But this classification does not fully characterize the affinity of CBMs on fibers [67]. Indeed, Jung, et al. [90] found the binding of the intact cellulases and corresponding CDs to bacterial microcrystalline cellulose was irreversible in all regions: Langmuir binding (region I), interstice penetration (region II), and interstice saturation (region III), but the CBMs bind reversibly in the region I. Therefore, the reversibility of the binding of different types of CBMs from different sources to different fibrous substrates remains to be investigated [73]. The reversibility of the adsorption is very important for practical application.

In addition to the affinity to polysaccharide substrate, CBMs were found to have physical adsorption onto other substrates, e. g. lignin [91,92]. The role of CBMs in unproductive enzyme binding was revealed by the adsorption of CBM on lignin substrate that physical adsorption contributed mainly to the so-called non-productive adsorption [93,94]. We won’t go into details here.

### 3.2. Use AFM to Explore the CBM-Substrate Interactions

CBM-substrates interactions are very interesting. There are many kinds of research on the changes in the process of CBM-interactions, among which the Small Angle X-ray Scattering Analysis [96], Quartz crystal microbalance (QCM) [53] and Atomic force microscope (AFM) [97] can observe this effect from the microscopic structure. AFM experiments provide a unique ‘biophysical’ method for direct observing of plant cell wall surfaces and pretreated cellulose microfibers at nanoscale resolution and with low in situ sample perturbations [98]. The binding affinity between CBMs and substrates can be measured using the piconewtonian sensitivity [99]. Researchers used AFM to monitor the interaction between CBMs and substrates. Zhang, et al. [100] observed the binding activity of CBM3 to the poplar cell wall cellulose in real-time using CBM3-functionalized gold nanoparticles (GNPs). They followed it up by single-molecule recognition imaging directly using the CBM3-functionalized AFM tip to map out the binding across the plant cell wall surface. Peng, et al. [101] demonstrated the application of AFM to observe the swelling of single-crystal cellulose fibers in real-time. Later, Zhang, et al. [102] studied the binding kinetics of CBM3 molecules with crystalline cellulose fibers extracted from poplar cell walls using AFM. Different concentrations of free CBM3 molecules were added to the buffer solution and bound to the crystalline cellulose samples fixed on the AFM matrix. CBM molecules were observed to bind to cellulose efficiently and regularly during in situ AFM imaging. These data provide strong support for explaining the adsorption phenomenon.

Above experiments provided an in-depth understanding of the binding mechanism of CBMs and cellulose at the single-molecule level. It improves the basic knowledge on the nature of forces that control the interaction of cellulolytic enzymes with the cellulosic and non-cellulosic components of lignocellulosic surfaces. However, the technique of modifying the AFM tips is cumbersome, time-consuming, and the number of linked proteins is uncertain. Additionally, the modified tips require high uniformity of substrates, and there is a lack of standards for the applied force and requires a lot of repetition.

CBMs modified AFM tips also provide a theoretical basis for treating fibers in the papermaking process. Arslan [95] using AFM measured the nano-scale forces acting between the model cellulase and a set of lignocellulosic substrates with controlled composition. The three model substrates investigated were kraft (KP), sulfite (SP), and organosolv (OPP) pulped substrates. These substrates varied in their surface lignin coverage, lignin type, xylan, and content of acetone extractives. The results indicated that the overall adhesion forces of biomass to CBMs increased linearly with surface lignin coverage. Kraft lignin showed the highest forces among lignin types investigated, which is indicated in Figure 2d. Other cutting-edge techniques such as single-molecule Dynamic Force spectroscopy (SMDFS) [102] and Scanning Electron Microscopy (SEM) (Figure 2e) [89], were used in combination with AFM to monitor the recognition and adsorption of CBMs on the substrate.

### 3.3. Other Methods to Study CBM-Substrate Interactions

There are many other methods can be used to study the interactions between enzymes and substrate, like Hydrogen-Deuterium Exchange Mass Spectrometry (HDX-MS) [103], molecular dynamics simulations [104], and nuclear magnetic resonance (NMR) [105,106,107]. However, these research methods have not been used to study the force between CBMs and substrates, which should be a promising research direction in the future. Meanwhile, the visualization of CBMs-substrates interactions is also a good research direction [108,109].

## 4. Fiber Treatment Using CBMs

In recent years, with the boycott of plastic products, the demand for fiber materials has increased. However, due to the insufficient strength (especially wet strength) of packaging, paper straws, and the requirements for cleaner production, new biological treatment have gradually attracted the attention of researchers. Among them, application of single or multiple CBMs in fiber processing has extensively been utilized for improving the fiber properties. Treating cellulose fibers with CBMs can change their interfacial properties [110]. CBMs were fused to engineering enzymes/proteins for improved biological activity; or either used alone or conjugated with other reagents for enhanced wood and fiber treatment performance. Using CBM-based polymers to treat fibers to gain improvement of mechanical properties of fiber (secondary fiber) is an emerging area that should pay much attention [111].

### 4.1. Use CBMs Alone in Fiber Treatment

**Figure 3:**
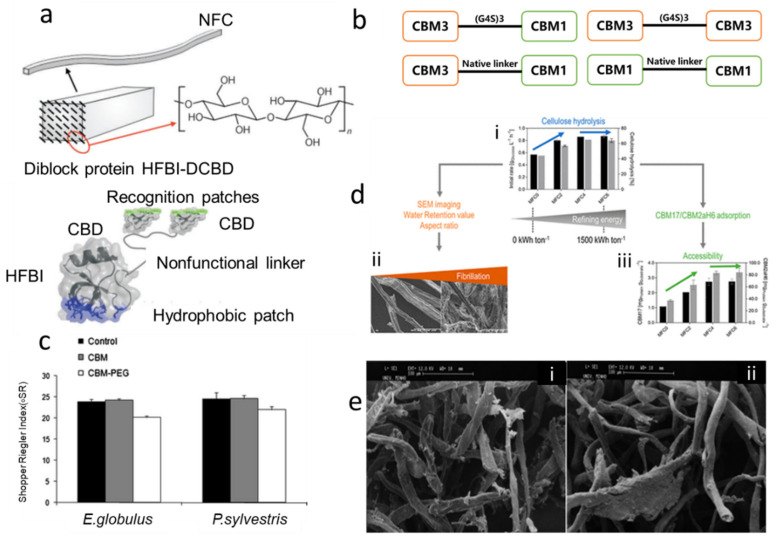
(**a**) A schematic presentation of the structure of the composite, reprinted from Ref [115] with permission from John Wiley and Sons. At the molecular level there are two functional blocks of the fusion protein amphiphilic hydrophobin−cellulose−binding domains (HFBI-DCBD) and its target surfaces; (**b**) Schematic structures of double CBMs, adapted from Ref [116]; (**c**) Shopper−Rieler Index of the *E. globulus* and *P. sylvestris* fibers treated with CBM, CBM-PEG and untreated, reprinted from Ref [67] with permission from Springer Nature; (**d**) (**i**) Impact of increasing refining energies on ease of enzyme−mediated hydrolysis of the microfibrillated cellulose (MFC) substrates. (**ii**) Impact of the refining energy on the fiber morphology. (**iii**) Impact of increasing refining energies on cellulose−binding module accessibility to the MFC substrates, reprinted from Ref [87] with permission from American Chemical Society; (**e**) SEM images of CF11 fibers treated with (**i**) and without (**ii**) CBD, reprinted from Ref [113] with permission from American Chemical Society.

Pala [112] first used separate CBM in papermaking to improve the water filtration and mechanical strength of secondary fiber paper. It showed that CBMs obtained by proteolysis of *T. reesei* cellulase can alter the drainage capacity of recycled pulp [113]. Shoseyov, et al. [114] and Laaksonen, et al. [115] developed biofunctional CBMs by genetic engineering and obtained paper-based materials with high mechanical strength. The adhesion domain was constructed by CBMs and amphiphilic hydrophobic protein (HFBI), see Figure 3a. A hydrophobic AFM tip can contact and lift a single fusion protein from the functionalized HFBI terminal through hydrophobic interactions between the tip surface and the HFBI hydrophobic patch [115]. Shi, et al. [116] constructed four recombinant CBMs, CBM3-GS(polypeptide (G_4_S)_3_)-CBM1, CBM3-NL(native linker from CBH1-1)-CBM1, CBM3-GS-CBM3, and CBM1-NL-CBM1, as shown in Figure 3b, the mechanical properties of paper were all enhanced. The folding resistance and tensile strength of paper increased by 27.4% and 15.5% after adding CBM3-GS-CBM3, and after the addition of CBM1-NL-CBM1, the paper tensile strength, elongation, and folding resistance was increased by 12.6%, 8.8%, and 16.7%, respectively. Among them, the improvement of tensile strength and folding resistance facilitate the use of containerboard paper. As shown in Figure 3e, the fiber agglomerations disappeared after CBMs treatment [113]. CBMs destroyed the aggregates dispersed on the larger fiber surface during drying. This is an interfacial phenomenon. CBMs treatment may reduce fiber interaction (fiber separation observed by SEM) through spatial and hydrophobic effects. Therefore, in the wet state, CBMs may have a better effect on fibers. However, the use of CBMs alone is expensive and cannot fufill are the desire requirements. Therefore, the researchers explored of the comination of other treaments along with CBMs to improve the fibers’ properties. Pretreated the fibers with CBMs and refining, then used water retention value (WRV), SEM, and aspect ratio to observe the change of the fiber. The results showed that using CBMs to more accurately conjecture enzyme accessibility, which is shown in Figure 3d, and it was found that refining did not significantly improve enzyme accessibility at the microfiber level of the cellulose substrate. Later, researchers began to study the conjugated additives, to achieve both performance and economic satisfaction.

### 4.2. CBMs Conjugated with Other Polymers for Fiber Treatment

CBMs can conjugate with other proteins or polymers because of their flexibility and specificity of CBMs. Protein side-chains contain many groups, such as amino, carboxyl, and hydroxyl groups [117]. Complex can be produced by common methods of blending (electrostatic attraction), and conjugation [118]. Many researchers began to construct conjugated systems of CBMs and polymers. CBM can be conjugated with various compounds such as polyethylene glycol (PEG), and anionic polyacrylamide (APAM) [119]. Machado [67] studied the adsorption of a CBM3 from the *Clostridium thermocellum scaffolding protein (Cip A)* to cellulose. The Carbohydrate binding domain-polyethylene glycol (CBM-PEG) module was constructed and the effect of this structure on the paper properties was studied (see Figure 3c). CBM-PEG improved the drainage capacity, but does not affect the mechanical properties of the paper which is due to the high water-binding capacity of PEG [120]. CBM-PEG improved the drainability of *E. globulus* and *P. sylvestris* pulps without affecting the physical properties of the paper [2]. Kitaoka and Tanaka [119] conjugated the CBM with APAM to improve the fiber binding, the results showed that both the dry tensile index and the wet tensile index were improved. However, both the fiber and the APAM are negatively charged, and the APAM is mostly used as a dispersant in the paper industry, in this case, there is still an improvement in mechanical properties, which can show the superiority of CBM for fiber binding.

The advantages of using independent CBM in fiber processing include the diversity of CBMs and avoiding the strength loss of using whole enzymes due to the catalytic activity of CD. More importantly, the fusion method with other polymers significantly reduces the amount of CBMs required and therefore reduces the costs. However, mass and economical production, preservation, and transportation of CBMs are still critical prerequisites for CBMs’ industrial applications. The current related work is very important because of the increased demand and performance requirements for paper products [121]. Further progress in this area is required to provide more environmentally friendly and more economical additives to improve fiber strength. Meanwhile, there are a few studies on the use of CBMs for nanocellulose materials, such as bacterial cellulose and microcrystalline cellulose materials [122]. This is also a major research direction because the structural properties of CBMs have the potential to alter the brittleness of nanocellulose materials [123]. Nanocellulose materials can be used in Pickering emulsions [124], ultrafiltration membrane [125,126] and paper straws [127].

### 4.3. Other Functions

In addition to the above effects on cellulose, the fusion of CBMs with other enzymes can also change biochemical characteristics and improve catalytic performance. And the CBMs of some thermophilic bacteria have high stability and belong to the thermostable domain. Studies have shown that fusion of thermostability domains to unstable protein domains can improve the thermostability of the latter [128,129]. Chhabra and Kelly [130] first reported the hyperthermophilic CBM fused to hyperthermophilic *endoglucanase*. The fusion protein was active on crystalline cellulose and the activity against microcrystalline cellulose was higher than that of the parent *endoglucanase* at 80 °C. Kavoosi, et al. [131] evaluated the impact of linker design on fusion protein production and performance. Liu, et al. [132] constructed an artificial bifunctional enzyme containing *carbonic anhydrase(CA)* from *Neisseria gonorrhoeae* and the CBM from *Clostridium thermocellum* with His6 tag, which can capture carbon dioxide from flue gas. As for the improvement of catalytic efficiency, Kittur, et al. [133] increased the catalytic activity of xylanase from *Thermotoga Maritima* for soluble xylan by fusion of CBM2. For optimizing the catalytic activity of *Cyclodextrin glycosyltransferase (CGTase)*. It is an important industrial enzyme for the production of cyclodextrins (CDs) from starch by intramolecular transglycosylation. *CGTase* of *Geobacillus sp.* was fused with the CBM20 of the *Bacillus circulans strain 251 CGTase* [134]. There seemed to be much room for improving its enzymological properties, such as improving its catalytic efficiency and substrate affinity, by replacing the domain of wild-type structural domain with a suitable CBM [135].

## 5. Conclusions and Outlook

CBMs are increasingly attracting attention as environmentally friendly biomass due to their unique properties such as wide distribution, small size, flexibility, stability, strong identification, strong plasticity, and the ability to fuse with enzymes or organisms to improve their functions. The application development in the paper industry, biomaterials, and other fields has extremely high commercial value. Based on the summary results of this review, the authors of this paper believe that more types of CBMs should be explored to address the practical application issues such as the problems of thermal stability and thermal resistance of CBMs derived from non-heat-resistant bacteria, cost, and yield. For example, studying the conjugation of CBMs with other polymers is a method to increase the yield and reduce the cost. It is also important to study the important role of CBMs in promoting enzyme-substrate binding and substrate-specific recognition.

CBMs will have wider applications due to their small molecular weight and high diversities [136]. For example, CBMs can be developed as a protein purification tag [137]. The low price of polysaccharides such as cellulose makes the use of them for adsorption columns an extremely valuable process. Due to CBM’s ability to bind specifically to insoluble substrates, it can be applied in medicine to create new recognition sites so that [138,139] CBM can bind to specific cell-surface polysaccharides and deliver drugs in a targeted manner [87,140]. In material design, previous studies showed how coupling engineered proteins containing CBMs as interlinking architectures with stiffer materials can tune the mechanical properties [123,141]. In general, although CBMs are small, they are of great value.
